# Therapeutic Effects of Intranasal Administration of Mesenchymal Stem Cell-Derived Secretome in Rats Exposed to Chronic Unpredictable Mild Stress

**DOI:** 10.3390/pharmaceutics17091129

**Published:** 2025-08-29

**Authors:** Alba Ávila, María Eugenia Riveros, Sofía Adasme, Coram Guevara, Rodrigo Del Rio, Fernando C. Ortiz, Nicole Leibold, Fernando Ezquer

**Affiliations:** 1 Centro de Medicina Regenerativa, Facultad de Medicina Clínica Alemana, Universidad del Desarrollo, Santiago, Av. Plaza 680, Las Condes 7610615, Región Metropolitana, Chile; aavilas@udd.cl; 2 Centro de Fisiología Celular e Integrativa, Facultad de Medicina Clínica Alemana, Universidad del Desarrollo, Santiago, Av. Plaza 680, Las Condes 7610615, Región Metropolitana, Chile; meriveros@udd.cl; 3 Departamento de Farmacología y Química Toxicológica, Facultad de Ciencias Químicas y Farmacéuticas, Universidad de Chile, Dr. Carlos Lorca Tobar 964, Santiago 8380494, Región Metropolitana, Chile; scadasmer@gmail.com; 4 Mechanisms of Myelin Formation and Repair Laboratory, Departamento de Biología, Facultad de Química y Biología, Universidad de Santiago de Chile, Santiago 9170022, Chile; coram.guevara@uautonoma.cl (C.G.); fernando.ortiz.c@usach.cl (F.C.O.); 5 Laboratory of Cardiorespiratory Control, Departamento de Fisiologia, Pontificia Universidad Católica de Chile, Santiago 7820436, Chile; rdelrio@kumc.edu; 6 Department of Cell Biology and Physiology, School of Medicine, University of Kansas Medical Center, Kansas City, KS 66160, USA; 7 Center for Aging Research and Healthy Longevity, Faculty of Sciences, Universidad Mayor, Camino la Piramide 5750, Santiago 8580745, Chile; 8 Department of Psychiatry and Neuropsychology, Mental Health and Neuroscience Research Institute, Maastricht University, Universiteitssingel 40, 6229 ER Maastricht, The Netherlands; nicole.leibold@maastrichtuniversity.nl

**Keywords:** depression, CUMS, mesenchymal stem cell, secretome

## Abstract

**Background**: Major depression is a significant source of suffering and economic loss. Despite efforts to understand this condition and find better treatments, the burden imposed by this disease continues to rise. Most approved pharmacological treatments for depression focus on controlling the availability of monoamines in synapses. However, accumulating evidence suggests that neuroinflammation, oxidative stress, and reduced hippocampal neurogenesis play key roles as causal factors in the development of major depression symptoms. Therefore, preclinical testing of pharmacological approaches targeting these factors is essential. Mesenchymal stem cells (MSCs) are known for their potential as powerful antioxidants and anti-inflammatory agents, exerting neuroprotective actions in the brain. They produce various therapeutic molecules in a paracrine manner, collectively known as secretome. **Methods:** In this work, we evaluated the antidepressant potential of repeated intranasal administration of MSC-derived secretome in an animal model of major depressive disorder induced by chronic mild unpredictable stress. **Results:** We observed that intranasal administration of MSC-derived secretome reduced the appearance of some of the behavioral parameters commonly associated with major depression, including anhedonic, apathetic, and anxious behaviors, inducing a strong reduction in the overall depression score compared to vehicle-treated animals. At the structural level, secretome administration prevented increased astrocyte density and the atrophy of astrocyte processes observed in vehicle-treated stressed animals. Additionally, secretome administration induced an increase in myelin levels and oligodendroglia in the cortex. **Conclusions:** Our data suggests that intranasal administration of MSC-derived secretome may represent a potential therapeutic alternative to current treatments for this devastating pathology.

## 1. Introduction

Suffering from major depression, characterized by symptoms such as a depressed mood, reduced interest in pleasurable activities, slowing down of thoughts, fatigue, feelings of worthlessness, recurrent thoughts of death, and suicidal ideation [1], significantly reduces the quality of life of patients and their close relatives. Therefore, this disease has been catalogued as one of the most debilitating mental illnesses [2]. It affects more than 350 million people worldwide [3], making it one of the leading causes of disability globally [2,4].

Pharmacological treatment for major depression currently focuses on regulating monoamine transmission. The first generation of antidepressant drugs consisted of monoamine oxidase inhibitors, which were later replaced by serotonin reuptake inhibitors such as fluoxetine. However, the efficacy of these treatments is far from optimal, as a large meta-analysis has shown only a modest difference between placebo and these antidepressant drugs [5].

The fact that drugs that increase synaptic levels of monoamines have some antidepressant effects led to the development of the monoamine hypothesis for depression [6]. This hypothesis posits that a deficiency in monoamine transmission, particularly in serotonin and noradrenaline, is the underlying physiological cause of the depressed state. However, this hypothesis fails to explain the time gap between the rapid antidepressant action at the molecular level (i.e., rapid increases in monoamine concentration) and the much slower reversal of symptomatology [7]. An alternative theory addresses this limitation, the neurogenesis hypothesis of depression, which suggests that the reduction of hippocampal neurogenesis due to chronic stress is a key factor in the induction of depression [8].

Supporting evidence for the role of reduced hippocampal neurogenesis in producing or maintaining depressive symptoms has been provided by studies showing that several antidepressants increase adult hippocampal neurogenesis [9,10]. Moreover, this neurogenic effect of antidepressants is essential for their efficacy, as inhibiting adult hippocampal neurogenesis revokes their clinical effects [11]. Interestingly, the timeline for the impact of these drugs on depressive symptoms mirrors their effect on hippocampal neurogenesis, supporting a possible causal link between both effects. Nevertheless, disruption of hippocampal neurogenesis by, for example, X-ray radiation of the hippocampus is insufficient to induce a depressive state and does not affect sensitivity to stress concerning depressive-like behavior [12], suggesting that depressive physiology is more complex and involves additional factors. One such potential factor is neuroinflammation. Various stressors activate inflammatory pathways. It has been reported that stressful psychosocial events induce adrenergic stimulation, which activates NF-κB, a potent inducer of inflammatory gene expression [13]. Moreover, chronic stress is a known fundamental contributor to the development of depression. Thus, the association between stress and inflammation suggests that inflammation could be a central component in linking stress to depression [14,15].

In general, inflammation is a protective response and a high-energy-demanding process. An inflammatory response is important for controlling infections, which are more likely to occur in conditions that induce stress. For example, in the case of stress caused by social interactions, a concomitant inflammatory state can be explained, in evolutionary terms, as an anticipatory response to a high probability of injury and subsequent infection. In fact, the relationship between psychosocial stress and inflammation appears to be strong, as childhood trauma, a significant risk factor for depression, is associated with a proinflammatory state in adulthood [16].

Inflammation is often accompanied by sickness behavior (e.g., prostration, reduced feeding, and decreased exploratory behaviors), which shares striking similarities with a depressed state. Therefore, it is reasonable to speculate that inflammation plays a contributory role in the depressed state, rather than simply coexisting with it. In line with this, intraperitoneal lipopolysaccharide (LPS) injection in rats induced a depressed state that correlated with LPS-induced alteration in the resting-state brain activity [17]. Moreover, in depressed patients, an inflammatory state was associated with anhedonia and decreased connectivity between the ventral striatum and the ventromedial prefrontal cortex (vmPFC) [18]. The analysis of resting-state functional magnetic resonance imaging data from depressed patients with varying levels of inflammation shows that inflammation contributes to network dysfunction and the associated symptomatology in depression [19]. However, currently available antidepressants primarily increase monoamine transmission and do not target inflammation or neurogenesis. Therefore, it is highly relevant to test the effect of anti-inflammatory and neurogenic agents in the treatment of depression.

Mesenchymal stem cells (MSC), an adult type of stem cell known as the “guardians of inflammation” [20], have emerged as a promising therapeutic tool for treating complex neurological diseases. When these cells are preconditioned through in vitro incubation with proinflammatory molecules, they produce a broad array of therapeutic molecules, including anti-inflammatory cytokines and neuroprotective factors, among others [21]. Due to their strong anti-inflammatory and neuroprotective potential, MSC transplantation or administration of conditioned media derived from these cells (known as secretome) has been successfully tested in both preclinical and clinical studies for treating various pathologies associated with neuroinflammation, including ischemic stroke [22,23], perinatal brain injury [24], perinatal asphyxia [25], amyotrophic lateral sclerosis [26], traumatic brain injury [27], experimental autoimmune encephalomyelitis [28], and chronic pain [29], among others.

Adipose tissue-derived MSCs, administered by intravenous injection, have recently been shown to reduce depressive behavior and inflammation in a chronic model of depression induced by stress in mice [30]. Nevertheless, there are safety concerns regarding the systemic administration of stem cells, including the risk of tumor induction, infection, cell aggregation, and thrombosis [27]. Therefore, to advance towards a clinically feasible approach, these promising results should be tested using a safer biodrug.

In this study, we used male rats subjected to a chronic unpredictable mild stress (CUMS) protocol for 8 weeks to induce a depressive state. Then, animals were treated non-invasively with weekly intranasal administrations of secretome derived from in vitro preconditioned human adipose tissue-derived MSC (hAD-MSC). To contextualize our preclinical model within clinical nosology, outcomes modeled by CUMS paradigms are most closely related to major depressive disorder (ICD-10: F32, F33) [31]. We observed that treated animals showed a significant reduction in their depression score compared to vehicle-treated animals. Secretome administration reversed anhedonic, apathetic, and anxious behaviors. These behavioral improvements were correlated with a decrease in stress-induced astrocyte activation and an increase in myelin levels and oligodendroglia cell density, suggesting that this biodrug holds antidepressant potential.

## 2. Materials and Methods

### 2.1. Animals

Thirty-six adult male Sprague Dawley rats were obtained from the Pontifical Catholic University of Chile. The rats weighed 190–210 g at the start of the 1% sucrose solution adaptation and approximately 280–300 g when beginning the CUMS protocol. Rats in the control group (non-stressed) were housed in pairs within cages that contained an enriched environment, including a red transparent plexiglass tube and shredded paper, to prevent low-grade “boredom” stress that can develop in barren or standard housing. Although traditionally considered neutral, such environments can elevate basal corticosterone levels and subtly decrease exploratory behavior over time [32]. Animals in the stress groups, however, were housed individually in standard conditions because adding environmental enrichment could have partially mitigated the stressors and confounded group differences. The only exception for the stress group was social crowding, which was used as a stressor. Food and water were provided ad libitum, except when deprivation was used as a stressor. A standard 12 h light/dark cycle (lights on from 6 am to 6 pm) was maintained. All procedures received approval from the Universidad del Desarrollo Animal Ethics Committee.

### 2.2. Outline of the Study

All rats were acclimatized to the facility for two weeks before initiating the protocol. Two days before starting the stress procedure, all rats underwent behavioral testing to obtain baseline performance measurements. Based on body weight and the sucrose preference test (SPT), rats were randomly assigned to one of two groups: the control group (*n* = 12), which was not subjected to stress, and the CUMS group (*n* = 24), which underwent the CUMS protocol. Every 7 days for 11 weeks, 1% sucrose consumption and body weight were measured. At week 8 of the protocol, all rats were tested for behavioral performance, and the CUMS rats were subdivided into two subgroups based on weight and baseline performance to ensure group homogeneity: CUMS + vehicle group (*n* = 12) and CUMS + secretome group (*n* = 12). The stress protocol continued for an additional three weeks, during which four intranasal doses (administered every five days) of either vehicle or secretome derived from preconditioned MSCs were given (see below). Seventy-two hours after the last secretome or vehicle administration, behavioral tests were performed. Twenty-four hours after the last behavioral test, rats were deeply anesthetized by inhalation of isoflurane vapors, and blood samples were collected via cardiac puncture. The animals were then immediately perfused with 180 mL of a washing solution composed of 0.4% dextrose, 0.8% sodium chloride, and 0.8% sucrose. After intracardiac perfusion, the brain was quickly isolated, and one hemisphere was rapidly dissected on ice. Samples of the prefrontal cortex (PFC) and hippocampus (HP) were collected and frozen at −80 °C for expression analysis of proinflammatory cytokines and neurotrophic factors. The other hemisphere was fixed in 4% paraformaldehyde (PFA) for 3 days, incubated in PBS containing 10% sucrose for 2 days, and subsequently 30% sucrose for 4 days at 4 °C before immunofluorescence analysis. A detailed experimental timeline is presented in Supplementary Figure S1.

### 2.3. Chronic Unpredictable Mild Stress (CUMS) Procedure

The CUMS procedure in this study lasted for 11 weeks. Each rat selected for the CUMS protocol was subjected to one stressor per day, chosen from a set of six stressors, which included: (i) wet bedding and cage tilt for 22 h; (ii) food and water deprivation for 22 h; (iii) stroboscopic light exposure for 8 h; (iv) social crowding within animals from the same treatment group for 22 h; (v) immobilization for 2.5 h; and (vi) immobilization for 2 h, plus stroboscopic light exposure for 2 h. Stressors were applied in a semi-random order to reduce predictability and ensure that consecutive stressors did not involve limited access to food and water.

### 2.4. Behavioral Tests

All behavioral tests were conducted between 8:00 and 14:00 h, recorded and analyzed using the ANY-maze video tracking system (version 6.35, Stoelting Co. Wood Dale, IL, USA).

*Sucrose preference test (SPT):* Two weeks before starting the stress protocol, rats were exposed to two glass bottles: one containing 150 mL of autoclaved tap water and the other 150 mL of 1% (*w*/*v*) sucrose solution, for 18 h over four consecutive days. This procedure familiarized the animals with this sweet solution and reduced neophobia. Sucrose intake was measured for 1 h once a week throughout the CUMS protocol. After 30 min of testing, the position of the bottles was swapped to eliminate consumption bias due to place preference. Consumption was calculated by comparing the bottle weight before and after the 1 h test. The intake was expressed as a percentage of sucrose preference using the formula: sucrose preference (%) = sucrose solution consumption (mL) × 100/total liquid intake (mL).

*Open field test (OFT):* The open field consisted of 100 cm × 100 cm base, 40 cm high, with a wooden inner part painted black. Rats were placed in the center, and their locomotor activity was recorded for 5 min using a video camera placed in a zenithal position. The OFT arena was cleaned with 60% ethanol before placing a new animal. The room was lit by a soft white light (50–60 lux). Testing began at 8 a.m., and no stressor was applied to the rats for at least 12 h before the test. The following outcomes were automatically scored: total distance traveled, time spent in the center, and thigmotaxis (tendency to remain near the walls).

*Coat state (CS):* Coat state was assessed as an indicator of grooming efficiency. The condition of the animal’s coat was visually inspected, and eight body parts were evaluated: head, neck, dorsal coat, ventral coat, genital region, forepaws, hindlegs, and tail. A score of 0 was assigned for a clean coat, and 1 for a dirty or disheveled coat in each of the eight areas. To ensure consistency, all the rats were assessed by the same pair of investigators in a blind manner.

*Sucrose splash test (SST):* A 10% sucrose solution was sprayed on the dorsal coat of the animals (cephalic, middle, and caudal area). This induced grooming behavior, and the rats were then placed back in their home cage and recorded for 5 min to analyze animal grooming activity. The test was performed under a red light. An investigator manually scored the following behavioral parameters: cumulative duration of grooming, number of grooming bouts, average duration of a single bout, and grooming bout patterns.

*Female urine sniffing test (FUST):* This test monitors hedonic behavior in male rodents. Rats were exposed to the smell of urine obtained from female rats in the estrous phase. The rat was placed inside its cage, and 1 h before starting the test, it was habituated to a sterile cotton-tipped applicator inserted in the grid of the cage. This test was performed under a red light and recorded for 3 min. The test consisted of three phases: (i) exposure to the cotton-tipped applicator wetted with 20 µL of water for 3 min during which sniffing duration was recorded; (ii) a 45 min rest period; and (iii) exposure for 3 min to a cotton-tipped applicator infused with 20 µL of pool fresh urine from female rats of the same strain in the estrous phase. Sniffing duration was recorded and measured.

### 2.5. Determination of Global Depression Score

To assess the depressive state of the rats, a global score was calculated, modified from Ardi et al. [33]. The control group’s behavioral test results were used to calculate the mean ± standard deviation. Cut-off points were determined by multiplying the standard deviation by 1, 2, 3, and 4. A value of 0 was assigned to results within ±1 standard deviation (indicating no change or depression), 1 for ±2 standard deviations, 2 for ±3 standard deviations, 3 for ±4 standard deviations, and 4 for results greater than 4 standard deviations, indicating a greater depressed state [33].

### 2.6. Isolation, Expansion and Characterization of Human AD-MSCs

hAD-MSCs were isolated from fresh subcutaneous adipose tissue samples obtained from liposuction aspirates (abdominal region) of patients undergoing cosmetic liposuction. All patients provided written informed consent at the Clínica Alemana, Santiago, Chile, as previously reported [34]. All protocols were approved by the Ethics Committee of the Medical Faculty, Clínica Alemana-Universidad del Desarrollo. After two subcultures, cells were characterized based on their adipogenic and osteogenic differentiation potential and by the expression of surface markers, as previously reported [34].

### 2.7. Preconditioning of hAD-MSCs and Secretome Generation

Human AD-MSCs (passage 3) were cultured in minimal essential medium (α-MEM, Gibco) supplemented with 10% fetal bovine serum (FBS; HyClone), and 0.16 mg/mL gentamicin (Sanderson Laboratory, Santiago, Chile) at 37 °C and 5% CO_2_. To enhance the production of antioxidant, anti-inflammatory, and neurotrophic factors, when cells reached a 70% confluence they were preconditioned by incubation with 10 ng/mL TNF-α and 15 ng/mL IFN-γ (R&D System, Minneapolis, MN, USA) for 40 h [35]. After preconditioning, cells were washed three times with phosphate-buffered saline (PBS) and incubated for 48 h with α-MEM without phenol red and FBS. The culture medium (secretome) was then collected and centrifuged at 400× *g* for 10 min to remove all detached whole cells. The supernatant was subjected to a second centrifugation at 5000× *g* for 10 min to remove cell debris. This process reduces contamination of the secretome with proteins released from cell rupture. After that, the secretome was filtered using 0.22 μm filters and concentrated 50 times (*v*/*v*) using 3 kDa cutoff filters (Millipore, Darmstadt, Germany). The concentrates were then washed with 15 mL of saline to remove all small molecules present in the culture medium and reconcentrated using the same filters. Consequently, after these concentration steps, the secretome contained only the molecules larger than 3 kDa, resuspended in saline [35]. The protein concentration was determined by the BCA protein assay kit (Thermo Scientific, Waltham, MA, USA) and the secretome was frozen at −80 °C until use.

### 2.8. Evaluation of mRNA Levels of Neuroprotective and Anti-Inflammatory Factors in Preconditioned hAD-MSCs

After preconditioning, total RNA was purified from hAD-MSCs using TRIzol (Invitrogen, Carlsbad, CA, USA). RNA isolated from naïve hAD-MSCs (same donor, no preconditioning) was used as a control. One microgram of total RNA was used to perform reverse transcription with MMLV reverse transcriptase (Invitrogen, Carlsbad, CA, USA) and oligo-dT primers. Real-time PCR was performed to amplify the mRNA of neuroprotective factors (BDNF, NGF, bFGF, and VEGF-a) and anti-inflammatory factors (IL-10, IL-5, TSG-6, and indoleamine 2-3 dioxygenase (IDO) using a SYBERGreen reaction kit (Roche, Basel, Switzerland) in a Light-Cycler 1.5 thermocycler (Roche, Basel, Switzerland). Relative quantifications were performed using the ∆∆CT method. The mRNA level of each target gene was normalized against the mRNA level of the house-keeping gene β-actin for each sample.

### 2.9. MSC-Secretome Intranasal Administration

Eight weeks after the initiation of the CUMS protocol, rats in the CUMS-vehicle and CUMS-secretome groups were anesthetized by intramuscular administration of ketamine (60 mg/kg) and acepromazine (4 mg/kg) and kept in a supine position on a thermic pad during and after intranasal delivery of saline (0.9% NaCl) or secretome, respectively. Using a pipet, 20 μL of the solution was administered 1–2 mm away from each nostril. The rat quickly absorbed the drop. Five minutes later, the same procedure was repeated for the other nostril, with four doses in each nostril over 40 min. A total volume of 160 µL, containing 25 μg of secretome proteins (derived from 1 × 10^6^ preconditioned MSCs), was administrated intranasally in four doses on days 57, 64, 71, and 74 of the CUMS protocol. As a control, 160 μL of saline (0.9% NaCl) was administered using the same procedure.

### 2.10. Immunohistofluorescense and Microscopy Image Analysis

The telencephalon was cut into 30 µm-thick frozen slices. Six coronal slices from the hippocampus (HP) and medial prefrontal cortex (mPFC) were analyzed from each group.

*Glial fibrillary acidic protein (GFAP) and ionized calcium-binding adaptor molecule 1 (Iba-1) double labeling:* Sections from the HP and mPFC were blocked with PBS containing 10% normal goat serum (NGS), 1% bovine serum albumin (BSA), and 0.3% Triton at room temperature for 1 h. Sections were then incubated with a rabbit polyclonal anti-Iba-1 antibody (1:400 dilution, Wako Pure Chem, Osaka, Japan) and a mouse monoclonal anti-GFAP antibody (1:500 dilution, Sigma-Aldrich, Darmstadt, Germany) overnight at 4 °C. After rinsing with PBS-Triton 0.1%, sections were incubated with an Alexa Fluor-488 donkey anti-mouse antibody (1:500 dilution, ThermoFisher, Waltham, MA, USA)and Alexa Fluor-594 donkey anti-rabbit antibody (1:400 dilution, ThermoFisher, Waltham, MA, USA) at room temperature for 2 h. After rinsing with TBS-Tween 0.1%, samples were incubated with DAPI (1:100 dilution, Sigma-Aldrich, Darmstadt, Germany). Images were acquired using a laser scanning confocal microscope (Spectral C2+). Three stratum radiatum sites of the CA1 region of HP and the area corresponding to the mPFC were scanned to assess astrocyte activation (GFAP expression) and microglia activation (Iba-1 expression).

*Myelin binding protein labeling:* Prefrontal cortex slices were prepared in PBS at 4 °C and permeabilized with 1% Triton X-100 and 4% NGS for 2 h. Immunostainings for Olig2 and MBP were performed using a rabbit anti-Olig2 antibody (1:500 dilution, Invitrogen, Carlsbad, CA, USA), and a chicken anti-MBP antibody (1:800 dilution, Invitrogen, Carlsbad, CA, USA), diluted in 0.2% Triton X-100 and 4% NGS. Slices were incubated with primary antibodies for 2 nights, followed by 2 h exposure at room temperature to secondary antibodies coupled to Alexa Fluor-555 (1:500 dilution, Invitrogen, Carlsbad, CA, USA) and Cyanine-based Fluorescent-405 (1:500 dilution, Sigma-Aldrich, Darmstadt, Germany). Confocal images from cingulate, limbic and infralimbic cortices were acquired using a ×20 (NA = 0.8; 1.5-μm z-step) with an LSM 710 confocal microscope (Zeiss). Images were processed using the NIH’s ImageJ Fiji software version 2.9.0 as previously described [36]. To analyze the MBP staining intensity, images were acquired using the same parameters of acquisition (laser power = 0.6, digital gain = 1170, offset = −1043, scanning velocity = 6, 1024 × 1024 resolution, 2 lines of averaging, and 16-bit image). The analysis was performed on Z-projections built from 16 stacks of 1.29 µm deep [voxel 0.415 × 0.415 × 1.297 um^3^]. Integrated optic densities were quantified using the threshold tool (B&W default option, ImageJ (v. 1.54g) after removing outliers (at 50%, process tool). The particle density of Olig2+ was calculated by dividing the number of particles (using the particle analysis tool, with a size range of 0–100 um^2^, and circularity 0.00–1.00) by the total area.

For all immunofluorescence experiments, negative controls were generated by omitting the primary antibodies.

### 2.11. Gene Expression Analysis

Frozen HP and PFC samples were collected in tubes containing 500 μL of TRIzol (Invitrogen, Carlsbad, CA, USA), and total RNA was isolated following the manufacturer’s instructions. One microgram of total RNA was used to perform reverse transcription with MMLV reverse transcriptase (Invitrogen, Carlsbad, CA, USA) and oligodT primers. Real-time PCR reactions were performed in a 10 μL final volume containing 50 ng cDNA, PCR Light Cycler-DNA Master SYBER Green mix (Roche, Basel, Switzerland), 3 mM MgCl_2_, and 0.5 μM of the primers to amplify the proinflammatory factors TNF-α, IL-1β, and IL-6 and the neurotrophic factors BDNF and NFG using a Light-Cycler 1.5 thermocycler (Roche, Basel Switzerland). Controls without reverse transcriptase were included to confirm that amplicons were derived from mRNA and not genomic DNA. Relative quantification was performed by the ΔΔCT method. The mRNA level of each target gene was normalized against the mRNA levels of the housekeeping gen β-actin of each sample.

### 2.12. Statistical Analysis and Sample Size Determination

Statistical analyses were performed using GraphPad Prism software version 6.01. For behavioral experiments, one-way ANOVA followed by Sidak’s post hoc tests was used. When only two groups were compared, statistical significance was determined using Student’s *t*-test. Significance was set at *p* < 0.05, and all data are presented as mean ±SEM. Full statistical analyses of each figure are presented in the corresponding figure legends. The sample size determination was based on the sucrose preference test, a primary readout of anhedonia and thus a proxy for depressive-like behavior in preclinical models. We expected the normal control group to exhibit a sucrose preference of approximately 80–90%, and stressed animals to show a reduced preference of about 60–65%, with a standard deviation of 10%. Using G*Power 3.1.9.2 for a two-tailed independent-samples *t*-test (difference between two independent means), with an expected mean of 80% in the treated/normal group and 60% in the stressed group, a standard deviation of 10%, alpha of 0.05, and power of 0.95, the required sample size was 7 rats per group. Considering strain-specific heterogeneity in stress susceptibility and an estimated 30% rate of stress resilience, we increased the planned sample size to 10 rats per group to preserve statistical power.

## 3. Results

### 3.1. hAD-MSC Preconditioning to Improve the Production of Neuroprotective and Anti-Inflammatory Factors

**Figure 1:**
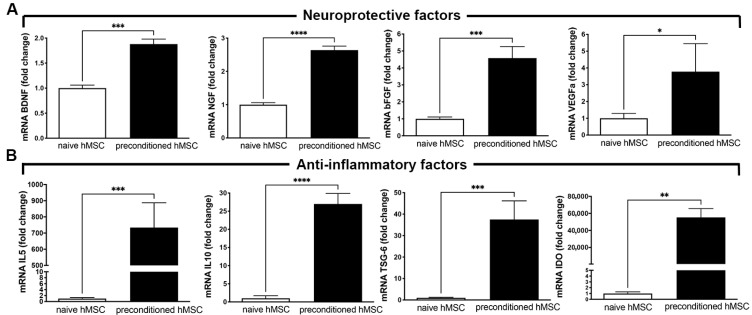
Preconditioning of hAD-MSCs to improve their therapeutic potential. (**A**) mRNA levels of neuroprotective factors BDNF, NGF, bFGF, and VEGF-a evaluated by RT-qPCR using total RNA obtained from hAD-MSCs after 40 h of preconditioning with the proinflammatory cytokines TNF-α and IFN-γ or vehicle (naïve MSC). (**B**) mRNA levels of anti-inflammatory factors IL-5, IL-10, TSG-6, and IDO evaluated in same samples as in (**A**). Data for each target gene was normalized against mRNA level of housekeeping gene β-actin in the same sample and presented as fold change in expression of preconditioned MSCs compared to naïve MSCs. Data are shown as mean ± SEM. *n* = 3 per experimental condition. (* *p* < 0.05; ** *p* < 0.01; *** *p* < 0.001; **** *p* < 0.0001 vs. naïve hAD-MSCs, Student’s *t*-test).

It has been reported that hAD-MSCs can produce a broad array of therapeutic molecules, and this paracrine potential can be further enhanced through preconditioning of the cells by in vitro incubation with proinflammatory cytokines, which increases the secretion of beneficial factors [35]. In agreement with this, we found that the preconditioning of hAD-MSCs with TNF-α and IFN-γ for 40 h significantly increased the mRNA levels of the neuroprotective factors BDNF, NGF, bFGF, and VEGF-a, compared to non-preconditioned (naïve) hAD-MSCs (*p* < 0.05 to <0.0001, Figure 1A). A similar trend was observed in the production of anti-inflammatory molecules, where the incubation of hAD-MSCs with proinflammatory cytokines greatly increased the mRNA levels of anti-inflammatory molecules including IL-5, IL10, TSG-6, and IDO, compared to non-preconditioned hAD-MSCs (*p* < 0.01 to <0.0001, Figure 1B). A more detailed description of the composition of the secretome derived from preconditioned MSCs is available in previous reports from our laboratory [37,38], particularly of its enrichment in proteins related to cell cycle control and proliferation, growth factors, and organization of the extracellular matrix, as well as to the immune response and lipid metabolism.

### 3.2. Neurovegetative and Behavioral Evaluations

Animals in both the control and CUMS groups started the experiment with comparable age and weight. However, after four weeks of stress, animals in the CUMS group began to show a reduced rate of body weight gain compared to the control group (*p* < 0.05). This difference in body weight persisted over the 11-week experimental period (*p* < 0.001) (Supplementary Figure S2).

### 3.3. Changes in Hedonic-Driven Behaviors

**Figure 2:**
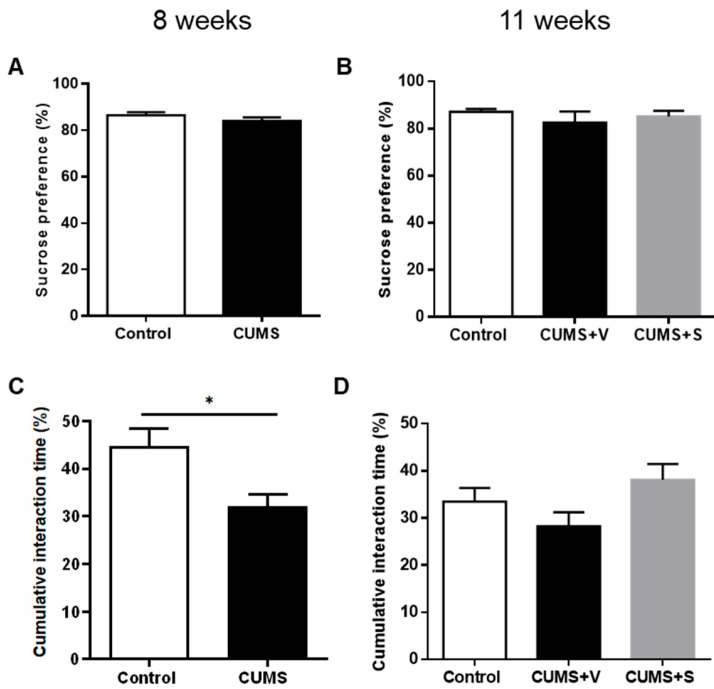
Anhedonia behavioral tests. (**A**) Sucrose preference (%) of rats subjected to 8 weeks of CUMS protocol and non-stressed animals (*n* = 12 control, *n* = 22 CUMS). (**B**) Sucrose preference of rats subjected to 11 weeks of CUMS protocol and treated with four intranasal doses of secretome derived from preconditioned hAD-MSCs or vehicle. Non-stressed animals were used as control (*n* = 12 control, *n* = 11 CUMS + Vehicle (CUMS + V), *n* = 11 CUMS + Secretome (CUMS + S)). (**C**) Female urine sniffing test: cumulative interaction time (%) with estrous phase urine of rats subjected to 8 weeks of CUMS protocol and non-stressed animals (*n* = 12 control, *n* = 22 CUMS). * *p* < 0.05, Mann–Whitney test. (**D**) Female urine sniffing test: cumulative interaction time (%) with estrous urine of rats subjected to 11 weeks of the CUMS protocol and treated with four intranasal doses of secretome derived from preconditioned hAD-MSCs or vehicle. Non-stressed animals were used as control (*n* = 12 control, *n* = 11 CUMS + Vehicle (CUMS + V), *n* = 11 CUMS + Secretome (CUMS + S). All data are expressed as mean ± SEM.

To assess the development of a reduction in the ability to experience pleasure, i.e., anhedonia, which is a central symptom in depressed patients, animals were tested in the sucrose preference test. In the CUMS group, after 8 weeks of stress, there was no significant reduction in the percentage of sucrose solution consumption relative to water when compared to the control group (Figure 2A). The intranasal administration of four doses of MSC-derived secretome did not result in a change in sucrose intake at week 11, as compared to the CUMS group treated with vehicle or the control animals (Figure 2B). In contrast, another hedonic behavior related to sexual motivation, i.e., the exploration of the scent of a sexually receptive female, assessed by the female urine sniffing test, was sensitive to the chronic stress exposure. CUMS animals showed a significant reduction in the time spent exploring the urine-damped cotton, indicating a reduction in sexual motivation compared to the control group (*p* < 0.05, Figure 2C). However, this reduction in sexual motivation did not reach statistical significance at week 11 due to the reduction in the number of animals in each experimental group. The time spent exploring the urine-damped cotton was greater in the secretome-treated CUMS animals compared to vehicle-treated CUMS animals, though this increase did not reach statistical significance (Figure 2D).

### 3.4. Changes in Self-Care-Related Outcomes

**Figure 3:**
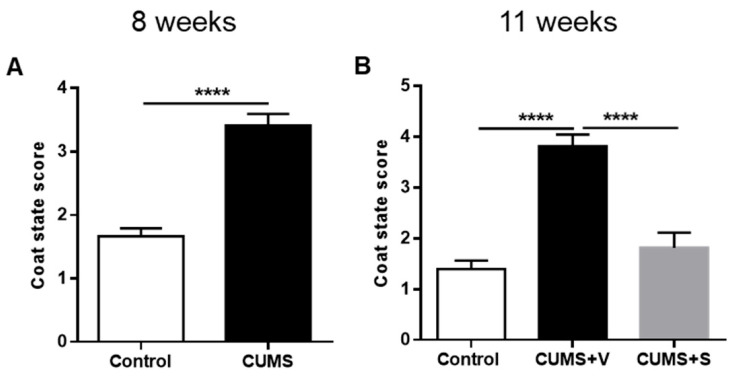
Coat status test. (**A**) Coat status score evaluating self-care behavior of rats subjected to 8 weeks of CUMS protocol and non-stressed animals (*n* = 12 control, *n* = 22 CUMS). **** *p* < 0.0001 vs. control; Mann–Whitney test. (**B**) Coat status score of rats subjected to 11 weeks of the CUMS protocol and treated with four intranasal doses of secretome derived from preconditioned hAD-MSCs or vehicle. Non-stressed animals were used as control (*n* = 12 control, *n* = 11 CUMS + Vehicle (CUMS + V), *n* = 11 CUMS + Secretome (CUMS + S)) **** *p* < 0.0001 vs. control and secretome treated animals, ANOVA with Sidak’s post hoc. All data are expressed as mean ± SEM.

**Figure 4:**
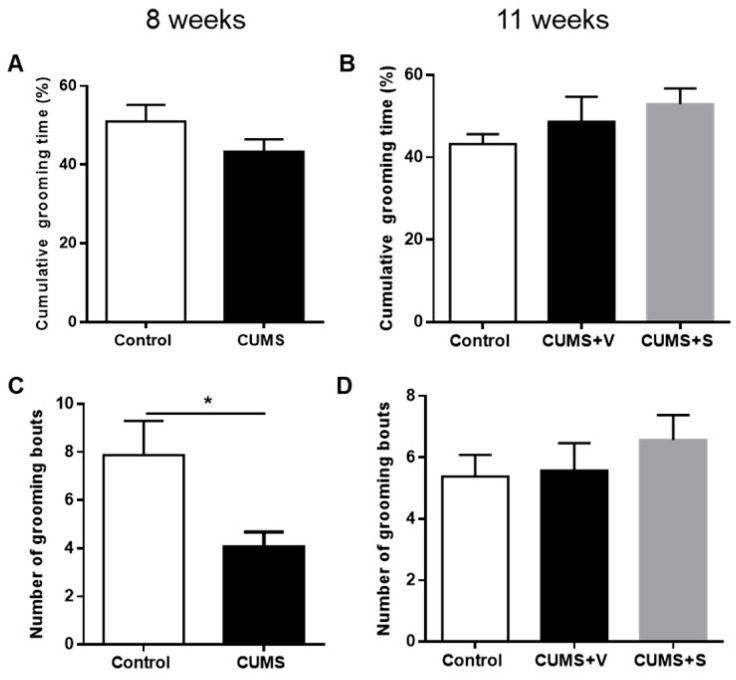
Grooming test. (**A**) Cumulative grooming time (%) and (**C**) number of grooming bouts of rats subjected to 8 weeks of the CUMS protocol and control non-stressed animals after spraying glucose solution on dorsal region to induce grooming behavior (*n* = 8 control, *n* = 14 CUMS). * *p* < 0.05 vs. control; Mann–Whitney test. (**B**) Cumulative grooming time (%) and (**D**) number of grooming bouts of rats subjected to 11 weeks of the CUMS protocol and treated with four intranasal doses of secretome derived from preconditioned hAD-MSCs or vehicle. Non-stressed animals were used as control (*n* = 8 control, *n* = 11 CUMS + Vehicle (CUMS + V), *n* = 11 CUMS + Secretome (CUMS + S) Mann–Whitney test. All data are expressed as mean ±SEM.

Healthy rats typically exhibit stereotypical grooming behavior, which can be negatively impacted by stress. This reduction in grooming behavior is often compared to the apathy and decreased self-care observed in depressed patients [39]. After 8 weeks of stress, rats in the CUMS group exhibited a marked deterioration in coat status, as indicated by a significantly higher score in the coat status evaluation (*p* < 0.0001, Figure 3A). Intranasal administration of four doses of MSC-derived secretome resulted in a reduction in the coat score of stressed animals when tested at 11 weeks. In contrast, the vehicle-treated stressed animals maintained their elevated coat score (*p* < 0.0001, Figure 3B). These results suggest that the MSC-derived secretome effectively mitigated alterations in coat condition associated with grooming behavior and/or other stress-related effects of stress on self-care. Further, grooming behavior in response to a sucrose solution spray applied on the dorsal region of the coat was also affected by chronic stress. Animals in the CUMS group showed significantly fewer bouts of grooming behavior during the 5 min observation period after the sucrose spray at 8 weeks (*p* < 0.05, Figure 4C). However, no significant differences in cumulative grooming time were observed between control and stressed rats (Figure 4A). Surprisingly, at 11 weeks, vehicle-treated stressed animals no longer exhibit a reduction in grooming bouts. Additionally, there was no effect of secretome administration on either the number of grooming bouts or cumulative grooming time (Figure 4D and 4B, respectively).

### 3.5. Changes in Anxiety-Related Outcomes

**Figure 5:**
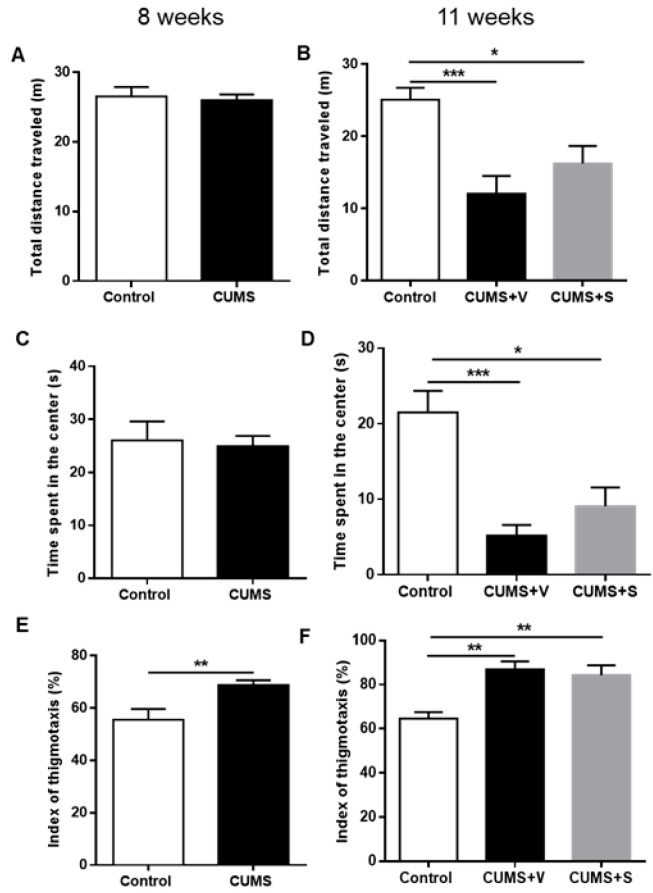
Locomotor activity in the open field test. (**A**) Total distance traveled, (**C**) time spent in the center, and (**E**) index of thigmotaxis as a measure of time spent near walls during five-minute test of rats subjected to 8 weeks of the CUMS protocol and control non-stressed animals (*n* = 12 control, *n* = 22 CUMS). ** *p* < 0.01, Kruskal–Wallis test. (**B**) Total distance traveled, (**D**) time spent in the center, and (**F**) index of thigmotaxis during five-minute test of rats subjected to 11 weeks of CUMS protocol and treated with four intranasal doses of secretome derived from preconditioned hAD-MSCs or vehicle. Non-stressed animals were used as control (*n* = 12 control, *n* = 11 CUMS + Vehicle (CUMS + V), *n* = 11 CUMS + Secretome (CUMS + S)). * *p* < 0.05; ** *p* < 0.01, *** *p* < 0.001; ANOVA with Sidak’s post hoc. All data are expressed as mean ±SEM.

When tested in the open field, after 8 weeks of stress, animals in the CUMS group did not show any significant difference in total distance traveled (Figure 5A) or time spent in the center (Figure 5C). However, CUMS animals exhibited a marked preference for staying near the walls of the arena (*p* < 0.01), a behavior known as thigmotaxis (Figure 5E). After 11 weeks of the CUMS protocol, vehicle-treated stressed animals showed a significant reduction in the total distance travelled compared to control non-stressed animals (*p* < 0.001, Figure 5B). Additionally, vehicle-treated CUMS animals spent less time in the center of the arena compared to control non-stressed animals (*p* < 0.001, Figure 5D), while intranasal secretome administration diminished both effects. The thigmotaxis index was higher in vehicle-treated CUMS animals compared to control animals, and secretome treatment did not reduce thigmotaxis behavior at 11 weeks (Figure 5F).

### 3.6. Global Score of Depression-Related Behavior

Major depression is a multifaceted disorder characterized by the simultaneous presence of a range of behavioral alterations, including emotional, neurocognitive, and neurovegetative symptoms [40]. To evaluate the overall impact of these symptoms and assess the therapeutic potential of antidepressive treatments, the use of a global score that incorporates all behavioral alterations into a single parameter has been proposed [41].

**Figure 6:**
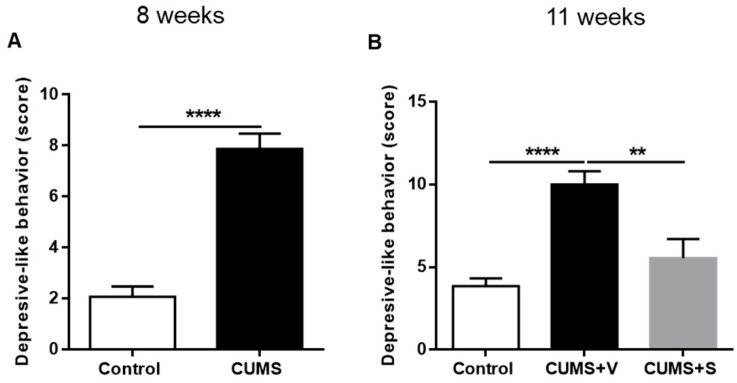
Global score of depression-related behavior. (**A**) Global depression score, including analysis of sucrose preference, total distance traveled, time spent in center in open field, % of interaction in FUST, and coat state score of rats subjected to 8 weeks of CUMS protocol and control non-stressed animals (*n* = 12 control, *n* = 22 CUMS). **** *p* < 0.0001 Kruskal–Wallis test. (**B**) Global depression score of rats subjected to 11 weeks of the CUMS protocol and treated with four intranasal doses of secretome derived from preconditioned hAD-MSCs o vehicle. Non-stressed animals were used as control (*n* = 12 control, *n* = 11 CUMS + Vehicle (CUMS + V), *n* = 11 CUMS + Secretome (CUMS + S). ** *p* < 0.01, **** *p* < 0.0001; ANOVA with Sidak’s post hoc test. All data are expressed as mean ± SEM.

Eight weeks after the initiation of the CUMS protocol, a significant increase in the depression-related behavior score was observed in the CUMS animals compared to non-stressed control animals (*p* < 0.0001, Figure 6A), indicating that the stressor combination effectively induces depressive-like behaviors. By 11 weeks of the CUMS protocol, vehicle-treated animals continued to show a significantly elevated depressive behavioral score (*p* < 0.0001, Figure 6B). However, intranasal administration of the MSC-derived secretome led to a significant reduction in the behavioral score, bringing it to levels comparable to those seen in the non-stressed control group (*p* < 0.01, Figure 6B), suggesting a potential antidepressive effect of the secretome.

### 3.7. Evaluation of Proinflammatory Factor Levels in the Frontal Cortex and the Hippocampus

To assess neuroinflammation in response to stress, we measured the mRNA expression levels of three key proinflammatory cytokines (IL-1*β*, IL-6, and TNF-*α*) in both the PFC and HP of the animals, 11 weeks following the initiation of the CUMS protocol. However, no significant differences in cytokine expression were observed between control and vehicle-treated stressed animals in both brain regions analyzed (Supplementary Figure S3A–F). Furthermore, secretome administration did not modify the expression levels of these interleukins in stressed animals (Supplementary Figure S3A–F).

### 3.8. Evaluation of Astrocytic Density, Microglial Density and Astrocyte Processes in the CA1 Region of the Hippocampus

**Figure 7:**
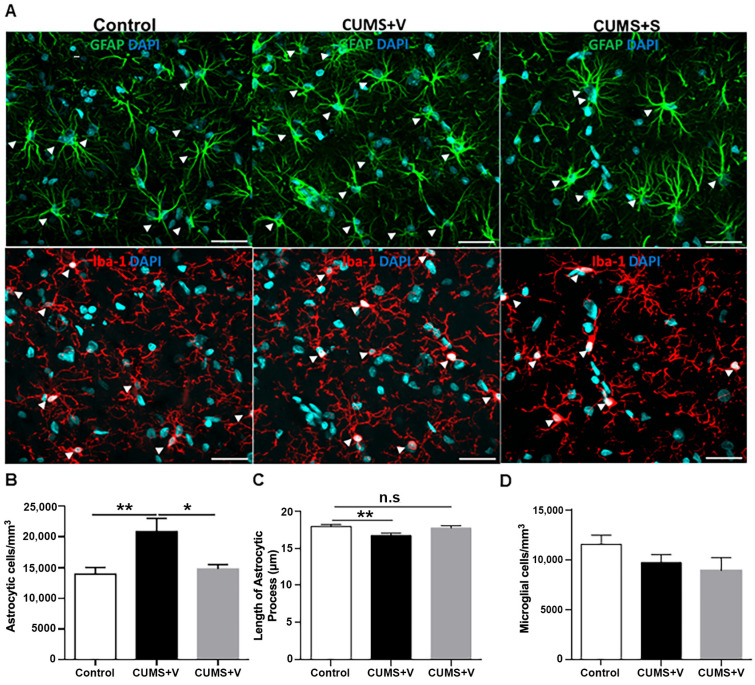
Analysis of astrocyte and microglial density and morphology in hippocampus. (**A**) Representative confocal microscopy images stained with GFAP (astrocyte marker; upper panel, green) and with Iba-1 (microglial marker; lower panel, red) from CA1 region of hippocampus of rats subjected to 11 weeks of the CUMS protocol and treated with four intranasal doses of secretome derived from preconditioned hAD-MSCs o vehicle. Non-stressed animals were used as control (*n* = 6 control, *n* = 6 CUMS + Vehicle (CUMS + V), *n* = 6 CUMS + Secretome (CUMS + S). Nuclei were counterstained with DAPI (blue). Scale bar = 100 µm. (**B**) Quantification of astrocyte density (GFAP^+^ cells) in CA1 region of the hippocampus. * *p* < 0.05, ** *p* < 0.01; ANOVA with Sidak’s post hoc test. (**C**) Quantification of length of primary astrocytic processes in CA1 region of hippocampus. ** *p* < 0.01; ANOVA with Sidak’s post hoc test. (**D**) Quantification of microglia density (Iba-1^+^ cells) in CA1 region of the hippocampus. Results are expressed as mean ±SEM. *n* = 6 per experimental condition. GFAP = Glial fibrillary acidic protein. Iba-1 = Ionized calcium-binding adapter protein. DAPI: 4′,6-diamidino-2-phenylindole. n.s. = non-significant.

Glial cells, including astrocytes and microglia, play an essential role in the brain’s response to injury, contributing to neuroinflammation and the disruption of neuroplasticity, exacerbating depression [42]. To evaluate changes in glial cell density and morphology, we conducted immunostaining for GFAP (astrocyte marker) and Iba-1 (microglial marker) in the CA1 region (stratum radiatum) of the hippocampus. We analyzed six representative sections from each group, using confocal microscopy. Results showed no significant changes in microglial density between the CUMS-treated group (vehicle or secretome) and control animals (Figure 7A,D). However, a significant increase in astrocytic density was observed in the hippocampus of CUMS animals treated with the vehicle compared to the control group (*p* < 0.01, Figure 7A,B). This increase in astrocytic density was completely reversed in stressed animals that received secretome treatment (*p* < 0.05, Figure 7A,B). Additionally, we assessed the morphology of astrocytes by measuring the length of their processes. In vehicle-treated stressed animals, astrocytes exhibited significantly shorter ramifications compared to control animals (*p* < 0.01, Figure 7A,C). However, astrocytes in secretome-treated stressed animals displayed a similar process length to those observed in the non-stressed control group (Figure 7A,C), indicating a restoration in astrocytic morphology following secretome treatment.

### 3.9. Myelination Evaluation

Previous reports suggest that changes in myelination are expected under chronic stress [43]. Therefore, we performed immunostainings of key brain regions related to depression, namely the cingulate, limbic, and infralimbic cortices, to study the degree of myelination. We carried out staining against the myelin binding protein (MBP). Additionally, to determine the density of the myelin-forming cells in these regions, we measured the expression of the Olig2 protein, a molecular marker for the entire oligodendroglia lineage [36,44,45].

**Figure 8:**
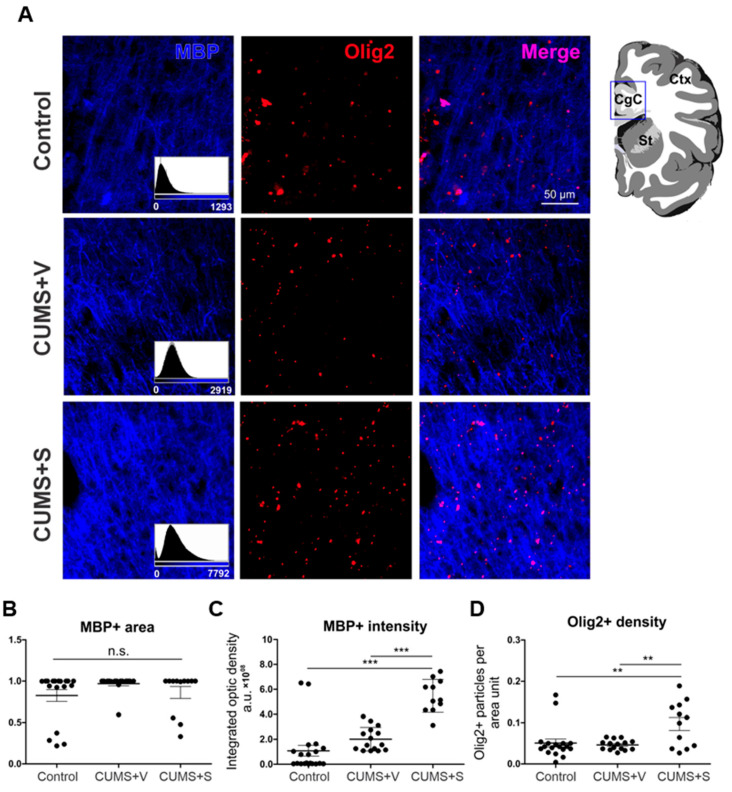
Analysis of myelin protein expression and oligodendroglial density. (**A**) Representative confocal microscopy images stained with MBP (myelin marker, blue) and Olig2 (oligodendroglial lineage marker, red) from cingulate cortex (CgC, inset in scheme, upper panels) of rats subjected to 11 weeks of CUMS protocol and treated with four intranasal doses of secretome derived from preconditioned hAD-MSCs o vehicle. Non-stressed animals were used as control (*n* = 6 control, *n* = 6 CUMS + Vehicle (CUMS + V), *n* = 6 CUMS + Secretome (CUMS + S). Note that MBP expression intensity (blue, lower panel) is increased in the secretome-treated group. To facilitate the comparison, the histogram of the raw intensity distribution for the same images is shown (inset in the MBP channel). Scale bar = 50 µm. (**B**) Quantification of MBP+ area as a fraction of total area. (**C**) Quantification of MBP intensity as integrated optical density. *** *p* < 0.001, ANOVA followed by Bonferroni’s Multiple Comparison Test. (**D**) Quantification of Olig2 particle density as Olig2+ particles per area. ** *p* < 0.01, ANOVA followed by Bonferroni’s Multiple Comparison Test. Results are expressed as mean ± SEM. *n* = 6 per experimental condition. MBP = Myelin basic protein. Olig2 = Oligodendrocyte transcription factor. n.s. = non-significant.

No significant differences were observed across the different brain regions within the experimental groups. Next, the data were pooled for all brain regions and compared between treatment groups. Interestingly, the myelinated area, measured as the area expressing MBP, did not differ between groups (Figure 8A,B). However, fluorescence intensity was significantly higher in those animals treated with the secretome (*p* < 0.001, Figure 8A,C). Consistently, the density of Olig2+ particles, an indirect measure of cells expressing Olig2, was also significantly increased under secretome treatment (*p* < 0.01, Figure 8A,D). Thus, these results suggest that secretome administration induced an increase in myelin production, likely due to the rise in the density of myelin-forming cells.

## 4. Discussion

The data from the current study show a beneficial effect of intranasal secretome administration on reducing some behavioral manifestations of stress-induced depressive-like behavior in adult male rats. Regarding the characteristics of the secretome, we confirmed the effectiveness of preconditioning in increasing mRNA levels of anti-inflammatory and neuroprotective factors. TNF-α/IFN-γ preconditioning is widely reported to potentiate the immunomodulatory and neuroprotective profile of MSCs and their secretome. At the protein level, this stimulus enhances indoleamine 2,3-dioxygenase (IDO) activity, prostaglandin E2 (PGE2), and TNF-stimulated gene-6 (TSG-6), alongside shifts in cytokine/chemokine output (e.g., attenuation of proinflammatory mediators such as TNF-α and IL-1β and context-dependent increases in IL-10, CXCL9/10, and CCL2). Collectively, these changes bias innate immune responses toward an anti-inflammatory, pro-resolving state [46,47]. Targeted protein-level assays and a multiplex cytokine/chemokine panels in preconditioned cells and their secretome are necessary to mechanistically link these pathways to the observed behavioral and neurobiological effects. However, consistent with our translational focus, we prioritized in vivo functional readouts in this study. Based on previously reported biodistribution assays for the exosome component of intranasally delivered secretome, we assume a widespread distribution across virtually all brain structures [48]. Acellular mesenchymal stromal cell (MSC) secretomes are anticipated to carry a lower alloimmunogenic burden than live-cell products, as they lack proliferative donor cells and MHC/co-stimulatory expression, yet retain the paracrine mediators that drive immunoregulation [49,50]. In line with this, we did not observe overt local inflammatory reactions or behavioral signs suggestive of acute immune intolerance following secretome administration (e.g., no injection-site swelling, grooming changes, or acute sickness behavior). While subclinical immune responses cannot be excluded, our data, together with prior literature using the same human preconditioned MSC-derived secretome administered intranasally, suggest a favorable tolerability profile for acellular secretomes [51].

The presentation of depressive symptoms in human patients is not uniform but rather a heterogeneous mixture of symptoms [52]. Depression subtypes have been identified, such as atypical, seasonal, or melancholic depression [53]. Like the heterogeneity of psychiatric symptoms, physiological alterations vary between patients, and hence the parameters affected and the direction of the alterations also differ [54]. This heterogeneity is difficult to incorporate into an experimental animal model. Although valuable contributions have been made to progress in this area [55,56,57], much work is still needed to validate each specific subtype model. Instead of using subtype profiles for depression models in rodents, a score that incorporates several behavioral parameters to evaluate the success of the model as a whole and the effect of a potential treatment could be a more suitable approach [58,59].

In line with this reasoning, we evaluated behaviors related to hedonism, self-care, and anxiety, and analyzed these with different tests. When these behaviors were first assessed separately, modifications in behaviors due to chronic stress exposure were significant for some, but not all, behaviors, and only at specific time points. This leaves questions about the sensitivity and specificity of individual behavioral assays. Future research, including an extended battery, is needed to better assess the therapeutic potential of the secretome in major depressive disorder. A similar scenario was observed regarding the effects of secretome treatment. One limitation of the present study is that we relied on the chronic unpredictable mild stress (CUMS) paradigm without biochemically verifying stress loading, since circulating corticosterone or other glucocorticoid readouts were not measured, leaving open the possibility that some animals were insufficiently stressed or had already habituated to the protocol, and therefore we could not confirm if all animals were comparably and sufficient stressed. Nevertheless, our protocol strongly reduced the animals’ weight gain over time, which is a reliable marker for the successful induction of chronic stress [60]. Therefore, the lack of expected changes in some behaviors after chronic stress exposure cannot be attributed to an insufficiently strong stress protocol. Consequently, we also analyzed the effect of each behavior as a contributor to an overall global depressed state score, which allows sub-profiles to vary between different animals (as observed in human patients). Using this approach, a robust effect appeared for chronic stress, which was fully reversed by secretome treatment, demonstrating its beneficial effect.

While the existence of different depression subtypes in patients is generally accepted, it remains arguable whether different profiles are related to different mechanisms of depression induction. In humans, for example, sexual hormone alterations, chronic illness, and childhood trauma are different factors that could activate different mechanisms, and hence also lead to different symptom profiles [61,62,63]. In the present study, to induce a depressed-like state, all rats were exposed to the same combination of stressors, based on which the same symptom profile and behavioral performance were expected. Nevertheless, it is very common to observe interindividual differences between animals. Subtle genetic differences can explain these differences [64] and slight variations in stress experience. For example, being the first or last animal to be handled for a specific stress manipulation could lead to different amounts of anticipation of stress experience or stress contagion [65].

When looking at the behavior tests separately, rather than as part of the global depression score, we did not observe a clear effect of secretome administration on the expected reversion of anhedonic behavior. Chronically stressed animals receiving intranasal vehicle or secretome administration showed a spontaneous reversion in the reduction of sexually motivated scent exploration in the FUST. Furthermore, no decrease in sucrose preference was observed either at 8 weeks (after stress but before treatment) or at 11 weeks (after vehicle or secretome administration). Nevertheless, in the test of sexual motivation, the secretome treatment induced an increase in the time the animals explored the urine-dampened cotton. This increase suggests that it is unlikely that secretome treatment interferes negatively with libido, which is promising for the potential use of this biodrug as an antidepressant in humans. Sexual dysfunction, including decreased sexual desire, is frequently reported as a side effect of antidepressant treatment in humans, with about 63% of medicated depressed patients reporting sexual problems [66]. Among the drugs reported to be associated with these sexual alterations are the most used antidepressant drugs, particularly selective serotonin reuptake inhibitors [67], with men showing a greater reduction in sexual desire than women [68]. This makes the effect of secretome on increasing sexual motivation in male rats clinically relevant.

In the present CUMS protocol, chronic stress was effective in inducing a depressed-like state, manifested in apathetic behavior, supported by the significant increase in the coat state deterioration, which is an effective endpoint for evaluating a depressed-like state in the chronic unpredictable stress rodent model [69]. Importantly, secretome administration fully reversed stress-induced coat deterioration, which is an important indication of its potential as an antidepressant.

In the OFT, secretome administration also reduced the depressive-like behavior of CUMS animals, increasing the total distance travelled and the time spent in the center of the arena, showing anxiolytic potential. Comparable results of secretome administration were obtained by the knockdown of the inflammasome NLRP1, a protein complex of the nucleotide oligomerization domain (NOD)-like receptor (NLR) family that is involved in neuroinflammation. This knockdown prevented the chronic stress induction of anxiety and anhedonia [70]. In that same study, the authors showed that chronic stress was able to activate hippocampal NLRP1 inflammasome. Interestingly, NLPR1 activation in response to stress promoted the release of the proinflammatory cytokines IL-1β, IL-18, IL-6, and TNF-α. As we reported here, the preconditioning of hAD-MSCs with TNF-α and IFN-γ for 40 h increased the production of anti-inflammatory and neuroprotective factors, which could counteract the stress-induced activation of NLRP1 and consequent neuroinflammation. This could represent a potential mechanism for the observed secretome antidepressant effects in the CUMS model, which deserves further exploration.

Recent evidence links neuroinflammation to mental disorders such as depression, as well as symptoms like impulsiveness, aggression, and feelings of helplessness, all of which are interconnected and positively associated with inflammation [15,71,72,73]. Tackling the neuroinflammatory response associated with stress and depression could, therefore, be key in preventing suicidal ideation and reducing the morbidity and mortality associated with mental health. Moreover, a heightened proinflammatory state (as shown by increased C-reactive protein levels) is associated with treatment resistance in depression [74], which again emphasizes the importance of further research into anti-inflammatory treatments as a promising approach to improving treatments for depressed patients, particularly those who are resistant to currently available options.

In our animal model, when combining the behavioral manifestations of chronic stress with molecular analyses of brain tissue, no associations were found. Specifically, neither mRNA levels of the three main proinflammatory cytokines in the PFC and HP of the animals, nor the levels of neuroprotective factors BDNF or NGF in the HP were altered. Our results are unexpected because it has been previously reported that increased BDNF levels are relevant for mediating the antidepressant effects induced by drugs with different targets and lifestyle factors [75,76]. However, it should be considered that levels of molecules such as the proinflammatory cytokines, BDNF and NGF can indicate different situations, as it is necessary to consider the balance between proinflammatory and anti-inflammatory molecules to understand the underlying process. Additionally, secretome administration could supply exogenous BDNF, NGF, and anti-inflammatory molecules directly [77,78], reducing the need for a compensatory rise in endogenous transcription while still promoting remyelination and behavioral recovery. Furthermore, the microRNAs contained in the secretome [79] could have suppressed translation or accelerated decay of cytokine messenger RNAs without changing their steady-state abundance; thus, functional cytokine output could have dropped in secretome-treated animals even while transcript counts remained unchanged. Finally, astrocytes and neurons locally translate mRNAs in end-feet or dendritic compartments [80]; when whole-tissue homogenates are analyzed, these focal changes may have been diluted below detection thresholds. Therefore, to disentangle whether and how the secretome’s anti-inflammatory action operates in the brain to reduce depressed behavior, Western blot analysis of a broader spectrum of anti-inflammatory mediators, as well as local cytokine expression in discrete brain regions, should be performed.

In contrast, an unequivocal marker of the development of neuroinflammation at the hippocampal level is the alteration in the number or structural phenotype of astrocytes [81]. When evaluating the structure of astrocytes in the CA1 region of the hippocampus, we observed that in the hippocampi of rats subjected to stress and treated with a vehicle, the density of astrocytes was significantly increased and their primary processes atrophied, which is consistent with the response to neuroinflammation [82]. While in the group that received secretome treatment, no significant differences were observed in the density of astrocytes or the length of the processes compared to the control non-stressed animals. This result is in line with results obtained using direct administration of secretome derived from non-preconditioned human bone marrow-derived MSCs into the dentate gyrus, which significantly increased both the number and length of astrocytic processes [83]. Considering this result in astrocytes, we could speculate that the intranasal administration of secretome, due to its high content of anti-inflammatory molecules, may counteract the neuroinflammatory process caused by stress in astrocytes. However, future studies should corroborate the putative anti-inflammatory actions of the secretome by quantifying GFAP and IBA-1 protein levels via Western blotting, and additional analysis of other neuroinflammatory markers is needed to gain a more comprehensive understanding of the neuroinflammatory process induced by chronic stress and its possible reversal by secretome treatment.

Regarding myelination, our results strongly suggest that secretome administration induced an increase in myelin production with no effect on the areas to be myelinated (i.e., no change in the areas expressing MBP). Nevertheless, the observed indications of enhanced myelination must be verified by Western blot assessment of MBP and Olig-2 expression. Along with these findings, we observed that secretome administration induced an increase in the population of Olig2-expressing cells, encompassing the entire oligodendroglial lineage. This is interesting from the mechanistic perspective, as it appears that the treatment enhanced the ability to produce myelin by increasing the number of cells capable of doing so, without modifying the regions of myelination. Our data is consistent with previous reports showing highly controlled oligodendroglial density and myelin production in different scenarios in the adult brain [84,85]. Since oligodendrocyte precursors (OPC) are unable to produce myelin, a likely scenario is that the secretome induced an increase in the density of mature myelinating oligodendrocytes, which, in turn, produced more myelin. However, the cellular mechanisms underlying the phenomena we described are beyond the scope of the present report, so further investigation is needed to address this interesting question.

Putting the present results into perspective, it is important to acknowledge the limitations of performing the experiments exclusively in male animals. This choice was made to allow some simplification in a complex model. However, given the higher prevalence of depression in women, and considering that the immune response in female is modulated by sex hormones and predisposes them to a higher inflammatory response to stress [86], this limitation is noteworthy. Since MSC-derived secretome is proposed to reduce inflammatory responses, an even greater therapeutic effect might be expected in females compared to males. Nevertheless, this issue warrants further investigation, incorporating female subjects. Additionally, our assessment window was designed to capture near-term outcomes following intranasal secretome administration within the CUMS paradigm, but it does not establish how long behavioral and histological benefits persist once treatment ceases. Without extended follow-up, we cannot distinguish transient symptomatic improvement from sustained disease modification, nor can we delineate the temporal relationship between behavioral changes and putative mechanisms such as microglial modulation or neurotrophic support. Future studies should therefore incorporate longer post-treatment intervals with repeated behavioral assessments and scheduled tissue collection to evaluate the maintenance, waning, or rebound of effects, alongside longitudinal biomarker panels (e.g., cytokines, neurotrophic factors) to define mechanistic durability. These extensions will clarify dose-spacing requirements and inform translational planning for maintenance regimens. Additionally, assessing behavioral and tolerability outcomes in non-CUMS animals intranasally treated with the secretome could further clarify the secretome’s baseline effects independent of stress exposure.

Our findings should be interpreted within the scope of the CUMS paradigm, which models chronic stress and captures select depression-relevant domains but does not recapitulate the full heterogeneity of clinical major depressive disorder [87,88,89]. While chronic stress is a principal risk factor for depression, it is neither necessary nor sufficient in all cases, and rodent behavioral readouts map only partially onto human symptom clusters [89,90]. Accordingly, our results should be understood as evidence of stress-mitigating and depression-relevant effects rather than definitive antidepressant efficacy. Translational extrapolation should therefore be cautious and contingent on replication across stress models, inclusion of both sexes, biomarker alignment with targeted mechanisms, and, ultimately, validation in rigorously designed clinical studies consistent with clinical nosology [31].

Altogether, the data presented here show that intranasal treatment with secretome derived from preconditioned hAD-MSCs can reverse some depressive-like states, and therefore represents a potentially effective treatment for depression symptoms in humans. Secretome administration warrants further investigation into its potential role in treating patients for whom no effective pharmacological treatment alternatives are currently available.

## Data Availability

The original contributions presented in this study are included in the article and Supplemental Materials. Further inquiries can be directed to the corresponding author.

## Supplementary Materials

The following supporting information can be downloaded at: https://www.mdpi.com/article/10.3390/pharmaceutics17091129/s1, Figure S1: CUMS protocol timeline, Figure S2: Cumulative body weight gain, Figure S3: Expression levels of proinflammatory cytokines in frontal cortex and hippocampus.

## Abbreviations

The following abbreviations are used in this manuscript:

BDNF	Brain-derived neurotrophic factor
BSA	Bobine serum albumin
bFGF	Basic fibroblast growth factor
CS	Coat status
CUMS	Chronic unpredictable mild stress
DAPI	4′,6-diamidino-2-phenylindole
FBS	Fetal bovine serum
FUST	Female urine sniffing test
GFAP	Glial fibrillary acidic protein
h-AD-MSC	Human adipose-derived mesenchymal stem cells
HP	Hippocampus
Iba-1	Ionized calcium-binding adaptor molecule-1
IDO	Indoleamine 2-3 dioxygenase
IFN-γ	Interferon gamma
IL-1b	Interleukin 1b
IL-5	Interleukin 5
IL-6	Interleukin 6
IL-10	Interleukin 10
LPS	Lipopolysaccharide
MBP	Myelin binding protein
MSCs	Mesenchymal stem cells
NGF	Neural growth factor
NGS	Normal goat serum
OFT	Open field test
OPC	Oligodendrocyte precursor cell
PBS	Phosphate-buffered saline
PFA	Paraformaldehyde
PFC	Prefrontal cortex
SPT	Sucrose preference test
SST	Sucrose splash test
TNF-α	Tumor necrosis factor alfa
TSG-6	Tumor necrosis factor-stimulated gene 6
VEGF-a	Vascular endothelial growth factor a
vmPFC	Ventromedial prefrontal cortex

## References

[1] American Psychiatric Association (2022). Diagnostic and Statistical Manual of Mental Disorders.

[2] Vos T., Barber R.M., Bell B., Bertozzi-Villa A., Biryukov S., Bolliger I., Charlson F., Davis A., Degenhardt L., Dicker D. (2015). Global, Regional, and National Incidence, Prevalence, and Years Lived with Disability for 301 Acute and Chronic Diseases and Injuries in 188 Countries, 1990–2013: A Systematic Analysis for the Global Burden of Disease Study 2013. Lancet.

[3] Smith K. (2014). Mental Health: A World of Depression. Nature.

[4] Whiteford H.A., Degenhardt L., Rehm J., Baxter A.J., Ferrari A.J., Erskine H.E., Charlson F.J., Norman R.E., Flaxman A.D., Johns N. (2013). Global Burden of Disease Attributable to Mental and Substance Use Disorders: Findings from the Global Burden of Disease Study 2010. Lancet.

[5] Undurraga J., Baldessarini R.J. (2012). Randomized, Placebo-Controlled Trials of Antidepressants for Acute Major Depression: Thirty-Year Meta-Analytic Review. Neuropsychopharmacology.

[6] Schildkraut J.J. (1965). The catecholamine hypothesis of affective Disorders: A review of supporting evidence. Am. J. Psychiatry.

[7] Hirschfeld R.M. (2000). History and Evolution of the Monoamine Hypothesis of Depression. J. Clin. Psychiatry.

[8] Mahar I., Rodriguez Bambico F., Mechawar N., Nobrega J.N. (2014). Stress, Serotonin, and Hippocampal Neurogenesis in Relation to Depression and Antidepressant Effects. Neurosci. Biobehav. Rev..

[9] Micheli L., Ceccarelli M., D’Andrea G., Tirone F. (2018). Depression and Adult Neurogenesis: Positive Effects of the Antidepressant Fluoxetine and of Physical Exercise. Brain Res. Bull..

[10] Eliwa H., Belzung C., Surget A. (2017). Adult Hippocampal Neurogenesis: Is It the Alpha and Omega of Antidepressant Action?. Biochem. Pharmacol..

[11] Santarelli L., Saxe M., Gross C., Surget A., Battaglia F., Dulawa S., Weisstaub N., Lee J., Duman R., Arancio O. (2003). Requirement of Hippocampal Neurogenesis for the Behavioral Effects of Antidepressants. Science.

[12] Surget A., Saxe M., Leman S., Ibarguen-Vargas Y., Chalon S., Griebel G., Hen R., Belzung C. (2008). Drug-Dependent Requirement of Hippocampal Neurogenesis in a Model of Depression and of Antidepressant Reversal. Biol. Psychiatry.

[13] Bierhaus A., Wolf J., Andrassy M., Rohleder N., Humpert P.M., Petrov D., Ferstl R., von Eynatten M., Wendt T., Rudofsky G. (2003). A Mechanism Converting Psychosocial Stress into Mononuclear Cell Activation. Proc. Natl. Acad. Sci. USA.

[14] Slavich G.M., Irwin M.R. (2014). From Stress to Inflammation and Major Depressive Disorder: A Social Signal Transduction Theory of Depression. Psychol. Bull..

[15] Beurel E., Toups F., Nemeroff C.B. (2020). The Bidirectional Relationship of Depression and Inflammation: Double Trouble. Neuron.

[16] Baumeister D., Akhtar R., Ciufolini S., Pariante C.M., Mondelli V. (2016). Childhood Trauma and Adulthood Inflammation: A Meta-Analysis of Peripheral C-Reactive Protein, Interleukin-6 and Tumour Necrosis Factor-α. Mol. Psychiatry.

[17] Zhu X., Ji M.-H., Li S.-M., Li B., Mei L., Yang J.-J. (2019). Systemic Inflammation Impairs Mood Function by Disrupting the Resting-State Functional Network in a Rat Animal Model Induced by Lipopolysaccharide Challenge. Mediat. Inflamm..

[18] Felger J.C., Li Z., Haroon E., Woolwine B.J., Jung M.Y., Hu X., Miller A.H. (2016). Inflammation Is Associated with Decreased Functional Connectivity within Corticostriatal Reward Circuitry in Depression. Mol. Psychiatry.

[19] Yin L., Xu X., Chen G., Mehta N.D., Haroon E., Miller A.H., Luo Y., Li Z., Felger J.C. (2019). Inflammation and Decreased Functional Connectivity in a Widely-Distributed Network in Depression: Centralized Effects in the Ventral Medial Prefrontal Cortex. Brain Behav. Immun..

[20] Prockop D.J., Youn Oh J. (2012). Mesenchymal Stem/Stromal Cells (MSCs): Role as Guardians of Inflammation. Mol. Ther..

[21] Ezquer F., Morales P., Quintanilla M.E., Santapau D., Lespay-Rebolledo C., Ezquer M., Herrera-Marschitz M., Israel Y. (2018). Intravenous Administration of Anti-Inflammatory Mesenchymal Stem Cell Spheroids Reduces Chronic Alcohol Intake and Abolishes Binge-Drinking. Sci. Rep..

[22] Dabrowska S., Andrzejewska A., Lukomska B., Janowski M. (2019). Neuroinflammation as a Target for Treatment of Stroke Using Mesenchymal Stem Cells and Extracellular Vesicles. J. Neuroinflamm..

[23] Doeppner T.R., Herz J., Görgens A., Schlechter J., Ludwig A.-K., Radtke S., de Miroschedji K., Horn P.A., Giebel B., Hermann D.M. (2015). Extracellular Vesicles Improve Post-Stroke Neuroregeneration and Prevent Postischemic Immunosuppression. Stem Cells Transl. Med..

[24] Thomi G., Surbek D., Haesler V., Joerger-Messerli M., Schoeberlein A. (2019). Exosomes Derived from Umbilical Cord Mesenchymal Stem Cells Reduce Microglia-Mediated Neuroinflammation in Perinatal Brain Injury. Stem Cell Res. Ther..

[25] Farfán N., Carril J., Redel M., Zamorano M., Araya M., Monzón E., Alvarado R., Contreras N., Tapia-Bustos A., Quintanilla M.E. (2020). Intranasal Administration of Mesenchymal Stem Cell Secretome Reduces Hippocampal Oxidative Stress, Neuroinflammation and Cell Death, Improving the Behavioral Outcome Following Perinatal Asphyxia. Int. J. Mol. Sci..

[26] Mazzini L., Vescovi A., Cantello R., Gelati M., Vercelli A. (2016). Stem Cells Therapy for ALS. Expert Opin. Biol. Ther..

[27] Das M., Mayilsamy K., Mohapatra S.S., Mohapatra S. (2019). Mesenchymal Stem Cell Therapy for the Treatment of Traumatic Brain Injury: Progress and Prospects. Rev. Neurosci..

[28] Genc B., Bozan H.R., Genc S., Genc K. (2019). Stem Cell Therapy for Multiple Sclerosis. Adv. Exp. Med. Biol..

[29] Ren J., Liu N., Sun N., Zhang K., Yu L. (2019). Mesenchymal Stem Cells and Their Exosomes: Promising Therapeutics for Chronic Pain. Curr. Stem Cell Res. Ther..

[30] Huang J., Huang W., Yi J., Deng Y., Li R., Chen J., Shi J., Qiu Y., Wang T., Chen X. (2023). Mesenchymal Stromal Cells Alleviate Depressive and Anxiety-like Behaviors via a Lung Vagal-to-Brain Axis in Male Mice. Nat. Commun..

[31] ICD-10 Version: 2016. https://icd.who.int/browse10/2016/en#/F32.0.

[32] Mieske P., Hobbiesiefken U., Fischer-Tenhagen C., Heinl C., Hohlbaum K., Kahnau P., Meier J., Wilzopolski J., Butzke D., Rudeck J. (2022). Bored at Home?—A Systematic Review on the Effect of Environmental Enrichment on the Welfare of Laboratory Rats and Mice. Front. Vet. Sci..

[33] Ardi Z., Albrecht A., Richter-Levin A., Saha R., Richter-Levin G. (2016). Behavioral Profiling as a Translational Approach in an Animal Model of Posttraumatic Stress Disorder. Neurobiol. Dis..

[34] Oses C., Olivares B., Ezquer M., Acosta C., Bosch P., Donoso M., Léniz P., Ezquer F. (2017). Preconditioning of Adipose Tissue-Derived Mesenchymal Stem Cells with Deferoxamine Increases the Production of pro-Angiogenic, Neuroprotective and Anti-Inflammatory Factors: Potential Application in the Treatment of Diabetic Neuropathy. PLoS ONE.

[35] Quintanilla M.E., Ezquer F., Morales P., Santapau D., Berríos-Cárcamo P., Ezquer M., Herrera-Marschitz M., Israel Y. (2019). Intranasal Mesenchymal Stem Cell Secretome Administration Markedly Inhibits Alcohol and Nicotine Self-Administration and Blocks Relapse-Intake: Mechanism and Translational Options. Stem Cell Res. Ther..

[36] Ortiz F.C., Habermacher C., Graciarena M., Houry P.Y., Nishiyama A., Oumesmar B.N., Angulo M.C. (2019). Neuronal Activity in Vivo Enhances Functional Myelin Repair. JCI Insight.

[37] Huang Y.L., De Gregorio C., Silva V., Elorza Á.A., Léniz P., Aliaga-Tobar V., Maracaja-Coutinho V., Budini M., Ezquer F., Ezquer M. (2023). Administration of Secretome Derived from Human Mesenchymal Stem Cells Induces Hepatoprotective Effects in Models of Idiosyncratic Drug-Induced Liver Injury Caused by Amiodarone or Tamoxifen. Cells.

[38] De Gregorio C., Contador D., Diáz D., Cárcamo C., Santapau D., Lobos-Gonzalez L., Acosta C., Campero M., Carpio D., Gabriele C. (2020). Human Adipose-Derived Mesenchymal Stem Cell-Conditioned Medium Ameliorates Polyneuropathy and Foot Ulceration in Diabetic BKS Db/Db Mice. Stem Cell Res. Ther..

[39] Van Reekum R., Donald F.R.C.P.C., Stuss T.L., Ostrander R.N. (2005). Apathy: Why Care?. J. Neuropsychiatry Clin. Neurosci..

[40] Malhi G.S., Mann J.J. (2018). Depression. Lancet.

[41] Faries D., Herrera J., Rayamajhi J., Debrota D., Demitrack M., Potter W.Z. (2000). The Responsiveness of the Hamilton Depression Rating Scale. J. Psychiatr. Res..

[42] Yang X.-Y., Wang H.-Q., Wang Z.-Z., Chen N.-H. (2025). Linking Depression and Neuroinflammation: Crosstalk between Glial Cells. Eur. J. Pharmacol..

[43] Yang P., Gao Z., Zhang H., Fang Z., Wu C., Xu H., Huang Q.J. (2015). Changes in Proinflammatory Cytokines and White Matter in Chronically Stressed Rats. Neuropsychiatr. Dis. Treat..

[44] Sahel A., Ortiz F.C., Kerninon C., Maldonado P.P., Angulo M.C., Nait-Oumesmar B. (2015). Alteration of Synaptic Connectivity of Oligodendrocyte Precursor Cells Following Demyelination. Front. Cell. Neurosci..

[45] Ortolani D., Manot-Saillet B., Orduz D., Ortiz F.C., Angulo M.C. (2018). In Vivo Optogenetic Approach to Study Neuron-Oligodendroglia Interactions in Mouse Pups. Front. Cell. Neurosci..

[46] Noronha Nc N.D.C., Mizukami A., Caliári-Oliveira C., Cominal J.G., Rocha J.L.M., Covas D.T., Swiech K., Malmegrim K.C.R. (2019). Priming Approaches to Improve the Efficacy of Mesenchymal Stromal Cell-Based Therapies. Stem Cell Res. Ther..

[47] López-García L., Castro-Manrreza M.E. (2021). TNF-α and IFN-γ Participate in Improving the Immunoregulatory Capacity of Mesenchymal Stem/Stromal Cells: Importance of Cell–Cell Contact and Extracellular Vesicles. Int. J. Mol. Sci..

[48] Ezquer F., Quintanilla M.E., Morales P., Santapau D., Ezquer M., Kogan M.J., Salas-Huenuleo E., Herrera-Marschitz M., Israel Y. (2019). Intranasal Delivery of Mesenchymal Stem Cell-Derived Exosomes Reduces Oxidative Stress and Markedly Inhibits Ethanol Consumption and Post-Deprivation Relapse Drinking. Addict. Biol..

[49] Dabrowska S., Andrzejewska A., Janowski M., Lukomska B. (2021). Immunomodulatory and Regenerative Effects of Mesenchymal Stem Cells and Extracellular Vesicles: Therapeutic Outlook for Inflammatory and Degenerative Diseases. Front. Immunol..

[50] Drobiova H., Sindhu S., Ahmad R., Haddad D., Al-Mulla F., Al Madhoun A. (2023). Wharton’s Jelly Mesenchymal Stem Cells: A Concise Review of Their Secretome and Prospective Clinical Applications. Front. Cell Dev. Biol..

[51] Quezada M., Ponce C., Berríos-Cárcamo P., Santapau D., Gallardo J., De Gregorio C., Quintanilla M.E., Morales P., Ezquer M., Herrera-Marschitz M. (2024). Amelioration of Morphine Withdrawal Syndrome by Systemic and Intranasal Administration of Mesenchymal Stem Cell-Derived Secretome in Preclinical Models of Morphine Dependence. CNS Neurosci. Ther..

[52] Carragher N., Adamson G., Bunting B., McCann S. (2009). Subtypes of Depression in a Nationally Representative Sample. J. Affect. Disord..

[53] Juruena M.F., Bocharova M., Agustini B., Young A.H. (2018). Atypical Depression and Non-Atypical Depression: Is HPA Axis Function a Biomarker? A Systematic Review. J. Affect. Disord..

[54] Rice F., Riglin L., Lomax T., Souter E., Potter R., Smith D.J., Thapar A.K., Thapar A. (2019). Adolescent and Adult Differences in Major Depression Symptom Profiles. J. Affect. Disord..

[55] Willner P., Gruca P., Lason M., Tota-Glowczyk K., Litwa E., Niemczyk M., Papp M. (2019). Validation of Chronic Mild Stress in the Wistar-Kyoto Rat as an Animal Model of Treatment-Resistant Depression. Behav. Pharmacol..

[56] Payne J.L., Palmer J.T., Joffe H. (2009). A Reproductive Subtype of Depression: Conceptualizing Models and Moving toward Etiology. Harv. Rev. Psychiatry.

[57] Heinzmann J.M., Kloiber S., Ebling-Mattos G., Bielohuby M., Schmidt M.V., Palme R., Holsboer F., Uhr M., Ising M., Touma C. (2014). Mice Selected for Extremes in Stress Reactivity Reveal Key Endophenotypes of Major Depression: A Translational Approach. Psychoneuroendocrinology.

[58] Guilloux J.P., Seney M., Edgar N., Sibille E. (2011). Integrated Behavioral Z-Scoring Increases the Sensitivity and Reliability of Behavioral Phenotyping in Mice: Relevance to Emotionality and Sex. J. Neurosci. Methods.

[59] Kraeuter A.K. (2023). The Use of Integrated Behavioural Z-Scoring in Behavioural Neuroscience—A Perspective Article. J. Neurosci. Methods.

[60] Sequeira-Cordero A., Salas-Bastos A., Fornaguera J., Brenes J.C. (2019). Behavioural Characterisation of Chronic Unpredictable Stress Based on Ethologically Relevant Paradigms in Rats. Sci. Rep..

[61] Batt M.M., Duffy K.A., Novick A.M., Metcalf C.A., Epperson C.N. (2020). Is Postpartum Depression Different From Depression Occurring Outside of the Perinatal Period? A Review of the Evidence. Focus (Am. Psychiatr. Publ.).

[62] Thom R., Silbersweig D.A., Boland R.J. (2019). Major Depressive Disorder in Medical Illness: A Review of Assessment, Prevalence, and Treatment Options. Psychosom. Med..

[63] Vares E.A., Salum G.A., Spanemberg L., Caldieraro M.A., De Souza L.H., Borges R.D.P., Fleck M.P. (2015). Childhood Trauma and Dimensions of Depression: A Specific Association with the Cognitive Domain. Braz. J. Psychiatry.

[64] Lacerda-Pinheiro S.F., Pinheiro Junior R.F.F., De Lima M.A.P., Da Silva C.G.L., Dos Santos M.D.S.V., Teixeira Júnior A.G., De Oliveira P.N.L., Ribeiro K.D.B., Rolim-Neto M.L., Bianco B.A.V. (2014). Are There Depression and Anxiety Genetic Markers and Mutations? A Systematic Review. J. Affect. Disord..

[65] Carnevali L., Montano N., Tobaldini E., Thayer J.F., Sgoifo A. (2020). The Contagion of Social Defeat Stress: Insights from Rodent Studies. Neurosci. Biobehav. Rev..

[66] Angst J. (1998). Sexual Problems in Healthy and Depressed Persons. Int. Clin. Psychopharmacol..

[67] Atmaca M. (2020). Selective Serotonin Reuptake Inhibitor-Induced Sexual Dysfunction: Current Management Perspectives. Neuropsychiatr. Dis. Treat..

[68] Serretti A., Chiesa A. (2009). Treatment-Emergent Sexual Dysfunction Related to Antidepressants: A Meta-Analysis. J. Clin. Psychopharmacol..

[69] Hu C., Luo Y., Wang H., Kuang S., Liang G., Yang Y., Mai S., Yang J. (2017). Re-Evaluation of the Interrelationships among the Behavioral Tests in Rats Exposed to Chronic Unpredictable Mild Stress. PLoS ONE.

[70] Song A.Q., Gao B., Fan J.J., Zhu Y.J., Zhou J., Wang Y.L., Xu L.Z., Wu W.N., Wu W.N. (2020). NLRP1 Inflammasome Contributes to Chronic Stress-Induced Depressive-like Behaviors in Mice. J. Neuroinflam..

[71] Beurel E., Jope R.S. (2014). Inflammation and Lithium: Clues to Mechanisms Contributing to Suicide-Linked Traits. Transl. Psychiatry.

[72] Troubat R., Barone P., Leman S., Desmidt T., Cressant A., Atanasova B., Brizard B., El Hage W., Surget A., Belzung C. (2021). Neuroinflammation and Depression: A Review. Eur. J. Neurosci..

[73] Herzog S., Bartlett E.A., Zanderigo F., Galfalvy H.C., Burke A., Mintz A., Schmidt M., Hauser E., Huang Y.-Y., Melhem N. (2025). Neuroinflammation, Stress-Related Suicidal Ideation, and Negative Mood in Depression. JAMA Psychiatry.

[74] Chamberlain S.R., Cavanagh J., De Boer P., Mondelli V., Jones D.N.C., Drevets W.C., Cowen P.J., Harrison N.A., Pointon L., Pariante C.M. (2019). Treatment-Resistant Depression and Peripheral C-Reactive Protein. Br. J. Psychiatry.

[75] Björkholm C., Monteggia L.M. (2016). BDNF—A Key Transducer of Antidepressant Effects. Neuropharmacology.

[76] Xu D., Gao L.N., Song X.J., Dong Q.W., Chen Y.B., Cui Y.L., Wang Q. (2023). Enhanced Antidepressant Effects of BDNF-Quercetin Alginate Nanogels for Depression Therapy. J. Nanobiotechnol..

[77] Su Y., Xu C., Cheng W., Zhao Y., Sui L., Zhao Y. (2023). Pretreated Mesenchymal Stem Cells and Their Secretome: Enhanced Immunotherapeutic Strategies. Int. J. Mol. Sci..

[78] Wihadmadyatami H., Zulficar M.A., Herewati H., Karnati S., Saragih G.R., Aliffia D., Pratama D.A.O.A., Handayani N., Kustiati U., Tirtosari D.R. (2025). Neuroprotection Effect of Bovine Umbilical Mesenchymal Stem Cell-Conditioned Medium on the Rat Model of Alzheimer’s Disease Mediated by Upregulation of BDNF and NGF and Downregulation of TNF-α and IL-1β. Open Vet. J..

[79] Constantin A., Comarița I.K., Alexandru N., Filippi A., Bojin F., Gherghiceanu M., Vîlcu A., Nemecz M., Niculescu L.S., Păunescu V. (2022). Stem Cell-derived Extracellular Vesicles Reduce the Expression of Molecules Involved in Cardiac Hypertrophy—In a Model of Human-Induced Pluripotent Stem Cell-Derived Cardiomyocytes. Front. Pharmacol..

[80] Meservey L.M., Topkar V.V., Fu M. (2021). meng MRNA Transport and Local Translation in Glia. Trends Cell Biol..

[81] Zhao Y., Huang Y., Cao Y., Yang J. (2024). Astrocyte-Mediated Neuroinflammation in Neurological Conditions. Biomolecules.

[82] Saur L., Baptista P.P.A., Bagatini P.B., Neves L.T., de Oliveira R.M., Vaz S.P., Ferreira K., Machado S.A., Mestriner R.G., Xavier L.L. (2016). Experimental Post-Traumatic Stress Disorder Decreases Astrocyte Density and Changes Astrocytic Polarity in the CA1 Hippocampus of Male Rats. Neurochem. Res..

[83] Campos J., Guerra-Gomes S., Serra S.C., Baltazar G., Oliveira J.F., Teixeira F.G., Salgado A.J. (2020). Astrocyte Signaling Impacts the Effects of Human Bone Marrow Mesenchymal Stem Cells Secretome Application into the Hippocampus: A Proliferation and Morphometrical Analysis on Astrocytic Cell Populations. Brain Res..

[84] Hughes E.G., Orthmann-Murphy J.L., Langseth A.J., Bergles D.E. (2018). Myelin Remodeling through Experience-Dependent Oligodendrogenesis in the Adult Somatosensory Cortex. Nat. Neurosci..

[85] Hughes E.G., Kang S.H., Fukaya M., Bergles D.E. (2013). Oligodendrocyte Progenitors Balance Growth with Self-Repulsion to Achieve Homeostasis in the Adult Brain. Nat. Neurosci..

[86] Slavich G.M., Sacher J. (2019). Stress, Sex Hormones, Inflammation, and Major Depressive Disorder: Extending Social Signal Transduction Theory of Depression to Account for Sex Differences in Mood Disorders. Psychopharmacology.

[87] Willner P. (2017). The Chronic Mild Stress (CMS) Model of Depression: History, Evaluation and Usage. Neurobiol. Stress.

[88] Planchez B., Surget A., Belzung C. (2019). Animal Models of Major Depression: Drawbacks and Challenges. J. Neural Transm..

[89] Belzung C., Lemoine M. (2011). Criteria of Validity for Animal Models of Psychiatric Disorders: Focus on Anxiety Disorders and Depression. Biol. Mood Anxiety Disord..

[90] Czéh B., Fuchs E., Wiborg O., Simon M. (2016). Animal Models of Major Depression and Their Clinical Implications. Prog. Neuropsychopharmacol. Biol. Psychiatry.

