# Supplementary material for: Therapeutic Effects of Intranasal Administration of Mesenchymal Stem Cell-Derived Secretome in Rats Exposed to Chronic Unpredictable Mild Stress
Source: Pharmaceutics. 2025 Aug 29;17(9):1129. doi: 10.3390/pharmaceutics17091129 (PMC12472816; doi:10.3390/pharmaceutics17091129)
Supplement: Supplementary file 1 [file pharmaceutics-17-01129-s001.zip › pharmaceutics-3807870-supplementary.pdf]

pharmaceutics

Supplementary Material: Therapeutic effects of intranasal administration of mesenchymal stem cell-derived secretome in rats exposed to chronic unpredictable mild stress

Alba Ávila, María Eugenia Riveros, Sofía Adasme, Coram Guevara, Rodrigo Del Río, Fernando C. Ortiz, Nicole Leibold, Fernando Ezquer

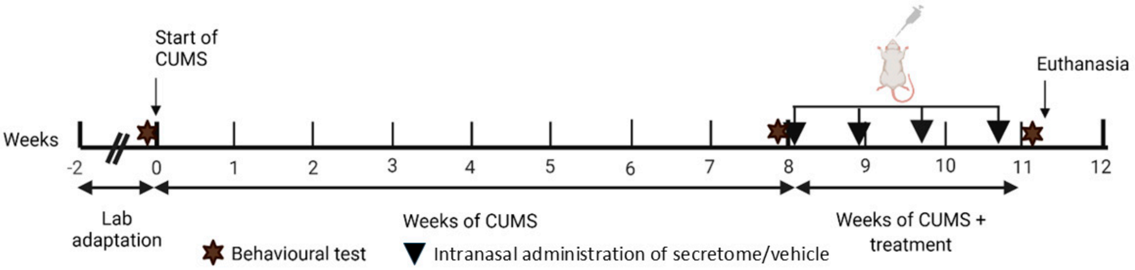

Scheme S1. Experimental Timeline.

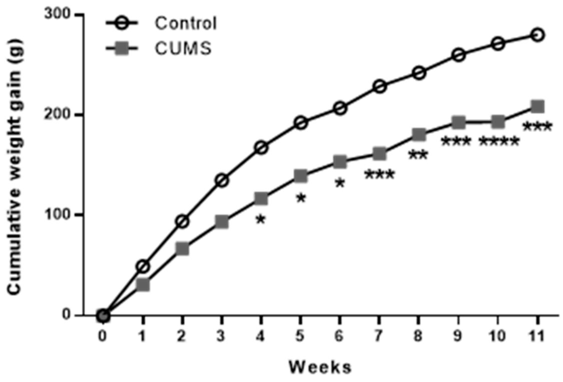

Supplementary Figure S2. Cumulative body weight gain. Rats submitted to the CUMS protocol gained less weight over time compared to control non-stressed animals. This effect was highly significant from four weeks onwards. n=12 control, n=22 CUMS. Results are expressed as mean ± SEM. ****p<0.0001, ANOVA with Sidak`s post-hoc test.

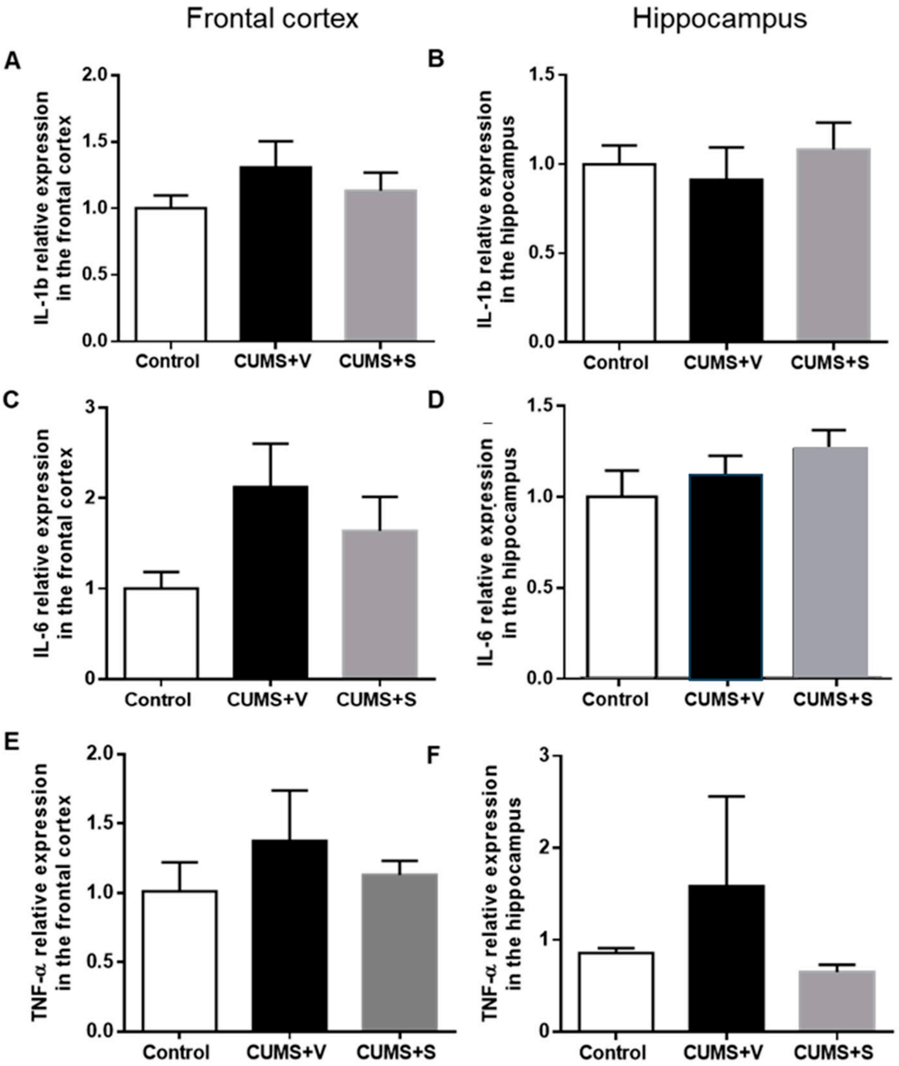

Supplementary Figure S3. Expression levels of pro-inflammatory cytokines in the frontal cortex and the hippocampus. A), B), C), D), E) and F) mRNA levels of the pro-inflammatory cytokines IL-1b, IL-6 and TNF-a determined by RT-qPCR in prefrontal cortex and hippocampus of rats subjected to 11 weeks of the CUMS protocol and treated with four intranasal doses of secretome derived from preconditioned hAD-MSCs or vehicle. Non-stressed animals were used as control. Expression levels were determined by the ΔΔCt method and normalized against b-actin expression in the same sample. Results are expressed as mean ± SEM. n=9 control, n=9 CUMS+Vehicle (CUMS+V) and n=9 CUMS+Secretome (CUMS+S).
